# Greedy 3-Point Search (G3PS)—A Novel Algorithm for Pharmacophore Alignment

**DOI:** 10.3390/molecules26237201

**Published:** 2021-11-27

**Authors:** Christian Permann, Thomas Seidel, Thierry Langer

**Affiliations:** 1 Department of Pharmaceutical Sciences, University of Vienna, Althanstrasse 14, 1090 Vienna, Austria; christian.permann@univie.ac.at (C.P.); thierry.langer@univie.ac.at (T.L.); 2 Inte:Ligand GmbH, Clemens Maria Hofbauer-Gasse 6, 2344 Maria Enzersdorf, Austria

**Keywords:** pharmacophore alignment, pharmacophore modelling, virtual screening, greedy algorithm, drug design

## Abstract

Chemical features of small molecules can be abstracted to 3D pharmacophore models, which are easy to generate, interpret, and adapt by medicinal chemists. Three-dimensional pharmacophores can be used to efficiently match and align molecules according to their chemical feature pattern, which facilitates the virtual screening of even large compound databases. Existing alignment methods, used in computational drug discovery and bio-activity prediction, are often not suitable for finding matches between pharmacophores accurately as they purely aim to minimize RMSD or maximize volume overlap, when the actual goal is to match as many features as possible within the positional tolerances of the pharmacophore features. As a consequence, the obtained alignment results are often suboptimal in terms of the number of geometrically matched feature pairs, which increases the false-negative rate, thus negatively affecting the outcome of virtual screening experiments. We addressed this issue by introducing a new alignment algorithm, Greedy 3-Point Search (G3PS), which aims at finding optimal alignments by using a matching-feature-pair maximizing search strategy while at the same time being faster than competing methods.

## 1. Introduction

3D Pharmacophores are spatial models that abstract the chemical features of molecules into labeled points [1,2,3]. These points can be supplemented by additional information like feature position tolerances or direction vectors. Such pharmacophore models have proven to be a useful tool for describing macromolecule–ligand interactions [4] and provide an intuitive abstraction of ligand features that are key for high binding-site affinity. Typically, pharmacophores modelling energetically favourable binding-site interactions are created by first finding two or more active molecules that bind to the target receptor of interest and then generating a consensus pharmacophore model. Alternatively, pharmacophores can also be derived directly from the 3D structure of one or more ligand–target complexes [2,5,6]. The latter approach generally leads to higher quality models due to the availability of bound-state ligand structures and binding-site environment information. In any case, the obtained pharmacophores usually need to be further refined by an expert user towards higher quality regarding their ability to discriminate between active and inactive molecules [2].

Since pharmacophore features model non-bonding interactions of functional groups and moieties without being tied to the underlying chemical structure, a single pharmacophore model may cover a wide variety of molecular structures with distinct scaffolds and functional groups that expose the same feature pattern. Pharmacophore-based techniques thus have an inherent scaffold hopping ability, which is of high value for lead optimization tasks or the tailored design of drug molecule [7].

One of the most prominent application of pharmacophores is their use as a filter for the virtual screening of large compound libraries (for which pharmacophores also have been pre-generated), which aims at finding molecules providing the same chemical feature pattern as specified by the query pharmacophore [5,8]. Found molecules where all or most of the query features match are likely to be also active towards the target of interest and represent a good first set of candidates for further experimental activity analyses. In comparison to a random selection of molecules, a refined set of candidate molecules significantly reduces resources spent with in vitro testing in the hit-finding phase and as a consequence also helps to reduce the overall drug discovery and development costs [9].

For virtual screening, query pharmacophore features are usually associated with certain positional tolerances to accommodate slight pharmacophore differences resulting from distinct molecular scaffolds and associated conformational spaces. How well two particular features match depends on how many feature pairs can be overlapped at most, given the optimal rotation and translation. The process of finding such an optimal rigid body transformation is called alignment. Pharmacophore alignment can be considered as the “heart” of the pharmacophore-based screening process in which the ultimate decision is made whether two pharmacophores match or do not match. Therefore, high robustness, reliability, and computational efficiency of the underlying algorithm are key factors regarding the usability of the screening software in real-world scenarios. Additional consideration has to be put on the conformational flexibility of molecules. Various methods for aligning molecules in a “flexible” way have been proposed [8], each with its benefits and downsides. In the context of pharmacophores, the problem of flexibility is often abstracted by simply pre-generating multiple low-energy conformations for a molecule and then aligning the pharmacophore of each conformer individually [8]. As pharmacophore-based alignments are relatively fast, large numbers of those alignments can be performed, which usually allows for a sufficiently good treatment of conformational flexibility. In the following, we do not go further into the details of molecule conformer generation since this topic is out of the scope of this study, and a wide variety of well-validated software tools for this task is already available [10,11,12,13,14].

For tackling the pharmacophore alignment problem, several methods and algorithms were devised [15,16,17]. Many of them have been implemented as part of well-known software platforms for pharmacophore modelling and screening like LigandScout [5,18], Phase [17,19], Catalyst [16,20], or MOE [21].

The algorithm by Wolber et al. [15] (due to the lack of a name, hereinafter referred to as the RM method/algorithm or RMM), for example, is used for generic pharmacophore alignment tasks and for pharmacophore matching in LigandScout’s virtual screening pipeline. In the first step of the algorithm, each pharmacophore feature gets abstracted through its feature type and a histogram of distances to the surrounding features. For each feature type present in the neighbourhood, one histogram is created, counting occurrences in the bins of the histogram. As distances may be close to falling into a neighbouring bin, smoothing filters are applied to the histogram. This results in the histograms of two features, where the features are in neighbouring bins, to be more similar. Next, a cost matrix for solving the pair assignment problem of the features of the two pharmacophores to align is created. This is done by assessing the similarity between the encoded features and setting a cost accordingly. This cost matrix is then used with the Hungarian algorithm [22] to find the best assignment that minimizes the overall cost. As a result, matching feature pairs are obtained, which are then used to compute a pharmacophore 3D alignment employing Kabsch’s method [23,24]. If the found alignment is not plausible according to the specified feature position tolerances, the pairs violating this condition are removed, and the method is repeated from the point of finding ideal feature pairs with the Hungarian method. If the alignment does meet the feature-matching criteria, the alignment transformation matrix is kept and applied to the underlying molecules. In a further post-processing step, the alignments are then checked whether they also fulfil any additional constraints imposed by directed features or exclusion volume spheres (see Section 2). Should those not be met, the alignment will be discarded and the algorithm proceeds until a valid alignment could be found, or it terminates without a found alignment solution.

Other alignment algorithms are based on volume overlap and model atoms or pharmacophore features such as Gaussian volumes, for which they optimize towards maximum overlap [25].

ROCS [26] is a well-known example for this group of methods, which considers Gaussian volumes defined by the atoms and (optionally) additional chemical feature information to align molecules.

Pharao [27] is also a method where pharmacophore features are modelled as Gaussian spheres. It operates by generating combinations of “feasible” pairs, which are then aligned by superimposing their coordinate centres and computing a rotation with a constrained gradient ascent. To create feasible pairs, the distance between two points of one pharmacophore and then the distance between two points (with matching feature types) of the other pharmacophore is measured. The difference between those distances is set in relation to the sum of distance thresholds for the four features and compared to a cut-off value ϵ (usually 0.5). If the value is smaller than ϵ, the pair is deemed feasible. Next, feasible pairs are assembled combinatorially, whereby any pair added to a combination must be feasible and compatible with all other pairs of the combination. A tuning of the ϵ parameter allows to reduce the total number of feasible combinations. For every combination produced, alignment is performed by first aligning the centres of the pharmacophore feature subsets. In the next step, constrained gradient ascent optimization is applied to find the rotation needed for the second pharmacophore to achieve maximum overlap with the first one. This procedure is carried out for four different starting orientations and results in a rotational transformation and an alignment score quantifying the overlap. Combinations with a larger number of matching features are aligned first, allowing for early termination if smaller combinations have a far smaller overlap.

**Figure 1:**
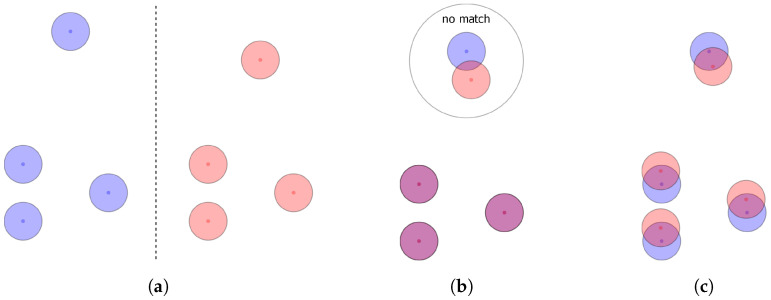
Illustration of different pharmacophore alignment strategies and the thus obtained results (**b**,**c**). For the sake of simplicity, pharmacophores (**a**) consist only of features of the same type. Different colours are just used to distinguish the pharmacophores. (**b**) Result from RMSD- or volume-based alignment. (**c**) Max. matching feature count-based alignment.

Existing alignment methods that optimize towards minimizing the root mean squared distance (RMSD) of corresponding feature pairs disregard that worse RMSDs may allow for more features to match. This disconnect between the optimization goal and the actual goal of alignment can be intuitively understood when thinking about the best case for an unambiguous alignment with RMSD. For any pair of pharmacophores, the best solution regarding this criterion would be to only align the three best matching features, resulting in a smaller RMSD than if one were to include even only one additional feature pair. For this reason, alignments may be discarded as the algorithm favoured the perfect fit of few features over the user defined model definitions. Methods optimizing for volume overlap of pharmacophore features modelled by Gaussian volumes have the same inherent problem since after optimization for maximum overlap, features may no longer fulfil positional and/or directional constraints and will be considered non-matching. This is an issue as the aim of the alignment, even for these algorithms, is to maximize the number of matching features. This problem affects the results of virtual screening drastically as the tested pharmacophore may be able to fit all of the features of a query pharmacophore but may still not be found due to the optimization goal. For illustration, Figure 1 shows two pharmacophore models that both consist only of features of the same type. Depending on the primary search goal—optimum RMSD/volume overlap vs. maximum matching feature count—different alignment results will be obtained (note that features are only considered matching if the central point of one feature is within the tolerance sphere of the other).

Another problem with the existing methods is how they deal with the allowed omission of user-defined less-critical “optional” features during pharmacophore alignment. The RM algorithm, for example, performs a combinatorial sampling and generates all possible combinations with #o missing features. If the models contain many features and #o is large, this will lead to an explosion of computational complexity and results in significantly longer runtimes. The generation of combinations based on omitted features and using them purely with Kabsch alignment additionally leads to another unintuitive issue: as the method may not identify a possible match without the omitted features due to the nature of purely optimizing towards RMSD, computing an alignment for less features may, by chance, find alignments where more of the features match. If aligning a larger number of features did not result in a valid alignment, less features are tried, and the centroid computed for the Kabsch alignment will be at a different place. With a different centroid, the algorithm may now be able to find an alignment for which the previously unmatched features match by coincidence. Thereby, allowing omitted features could lead to finding alignments that actually should have been found without them or even completely different alignments.

The treatment of exclusion volume spheres (see the methods section for details) is also problematic. Existing methods compute the alignments without considering these features and only discard the result post computation when they find exclusion volume constraints to be violated. This can lead to solutions, possibly even very good ones, to be discarded only because an exclusion sphere gets slightly touched. Visually, this would be an easy problem to solve, as just slightly moving a molecule may yield a valid alignment.

In summary, the core issue with all the discussed methods is that the optimization goal they follow is not in line with the intuition of pharmacophore models. As individual features have a positional tolerance threshold for matching, the best alignment solution may not be the one with minimal feature-pair RMSD or maximum volume overlap but the one providing the largest number of matched feature pairs under the given feature position and direction constraints. The latter criterion should be given preference especially when pharmacophore models are used as virtual screening filters in order to reliably identify all database molecules that provide a specific chemical feature pattern.

In the herein presented work, we address all of the above discussed main problems of the existing pharmacophore alignment methods by introducing a new alignment algorithm, Greedy 3-Point Search (G3PS), which aims at finding optimal alignments by using a matching feature pair maximizing search strategy while at the same time being faster than competing methods. The performance of the presented algorithm regarding its reliability in the identification of possible pharmacophore matches and its computational efficiency was assessed by screening several molecule datasets taken from the DUD-E database [28,29] with query pharmacophores derived from the corresponding ligand–target complexes. To provide an impression of the advances that could be achieved with our new algorithm, the obtained results are presented in direct comparison to the ones obtained with the RM algorithm.

## 2. Methods

This section provides a detailed description of our new alignment algorithm G3PS. Before presenting any details of the new algorithm, we first want to provide a definition of the used pharmacophore representation, introduce important concepts regarding the goodness of fit of two pharmacophore models given an optimal transformation, and point out additional aspects that are relevant for pharmacophore alignment.

### 2.1. Definitions and Concepts

**Pharmacophores.** Pharmacophores are a collection of labelled points representing chemical features [1,2,3]. Commonly used features include aromatic rings, hydrogen-bond acceptors/donors, and positive/negative ionizable and hydrophobic interactions, although others exist and custom features may be designed. For a given pharmacophore *A*, we denote feature *i* as $A_{i}$ for i=[1,2,…,|A|]. Features additionally contain a distance threshold $thresh(A_{i})$ indicating how far the centres of matching features may be away and, optionally, other restricting factors such as feature direction vectors.

**Exclusion Volume Spheres.** A pharmacophore *A* may contain additional features $A^{x}$, which can be interpreted as “anti-features.” These features restrict the space where atoms of the aligned molecule may occur, qualifying any alignment as invalid where atoms collide with such feature shapes. More generally, their goal is to abstract the presence of binding-site-environment atoms and model those locations, defining that there would not be enough space left for atoms of a binding molecule. For performance and simplicity reasons, these shapes are usually modelled as spheres, so we will use the terms “exclusion volume” and “exclusion sphere” synonymously. This greatly simplifies the evaluation of the validity of an alignment solution since just a check of the condition $dist\left(a,A_{i}^{x}\right)>thresh\left(A_{i}^{x}\right)$ for all atoms *a* and all exclusion spheres $A_{i}^{x}$ needs to be performed. If the condition is true for all combinations, the alignment is valid and can be kept.

**Fully Fitting Models.** For two models to fully fit, there must exist a transformation from pharmacophore *A* to *B* (and *B* to *A*) that includes only translation and rotation (no scaling) for which the following holds:

- There exists a mapping for all features of *A* and *B* where:

(1) All individual features map uniquely to a feature of the other model, creating index pairs (i,j).
(2) The labels (feature types) of paired features are equal $\left(i,j\right)\Rightarrow label\left(A_{i}\right)=label\left(B_{j}\right)$.
(3) The paired features distance is smaller than the larger distance threshold $\left(i,j\right)\Rightarrow dist\left(A_{i},B_{j}\right)<max\left(thresh\left(A_{i}\right),thresh\left(B_{j}\right)\right)$.

**Maximum Fitting Models.** Models with maximum fit may not meet the criterion of all features mapping to pairs, but all features of the query pharmacophore do. In other words, the pharmacophore that is used as a query will fully fit a subset of the other pharmacophore. This is the best achievable fit two models of different size can have. If the query pharmacophore is larger than the tested one, such a fit is not possible, and, at best, a partial fit can be achieved by allowing features to be omitted.

**Partially Fitting Models.** Models fitting partially are ones for which less than the number of features in the query pharmacophore match. To define the bound for a sufficient fit, a maximum number of omitted feature #o needs to be defined. If the best possible transformation, where the largest number of features match, is missing at most #o features of the query pharmacophore, the alignment is accepted, otherwise, the pharmacophores are considered non-matching.

**Quality of Model Fit.** The goal of aligning two pharmacophore models is to find a transformation that allows for the largest number of features to match. For this reason, the most important measure of quality is the number of matching pairs. A matching pair is defined as two features, one from each pharmacophore, for which all restricting properties are fulfilled, including having the same feature type and their distance being smaller than the larger distance threshold. If the number of matching feature pairs is equal for two found model alignment solutions, the quality of alignment can then be asserted by calculating the RMSD of the matched pairs. We want to put additional emphasis on the fact that the RMSD should only be considered regarding the alignment quality after determining the maximum number of matching features, as aiming for the smallest possible RMSD does not optimize towards finding the largest match. The best example for this is to only consider the best matching pair for computing the root mean squared distance. Any model (where at least one feature type matches) can achieve an RMSD of 0 if we only align one feature pair. By extension, a smaller number of matching features is more likely to have a small RMSD.

### 2.2. Greedy 3-Point Search (G3PS)

Our new alignment algorithm was implemented in a development version of Ligandscout [5,18] using the programming language Java and consists of two main parts followed by a post-processing step. First, the features of both pharmacophores are encoded and compared to identify which features of one pharmacophore are likely to match which features of the other pharmacophore. From this, first guesses are constructed using exactly three pairs of features, as this is the minimum required for computing an unambiguous 3D transformation. In the second step, iterative refinement is performed for each of the three-pair guesses. This refinement process collects additional pairs that do not break the alignment created from all previously collected pairs and applies transformations that optimize the alignment to better fit with the newly found pairs. Optionally, after this second step, exclusion volumes can be considered. Final additional checks can then be performed to filter out alignments for which more specific feature requirements are not met.

#### 2.2.1. Finding Three Pairs

To find a starting point for iterative refinement, at least three points need to be fixed in each of the pharmacophores, which allows to create an initial alignment. The number of such three-pair guesses being generated is an important factor for the overall runtime of the algorithm, as it defines how often refinement needs to be performed. Still, the downside of only having one or few starting alignments is that the globally optimal alignment may be missed. Therefore, we allow the user to define the maximum number of sampled guesses, enabling them to trade computation time for accuracy.

**Single Best Guess.** To compute an initial guess that is likely to result in a globally optimal matching, we computed a matrix describing how dissimilar each feature of one pharmacophore is to the features of the other. The creation of this dissimilarity matrix is performed by encoding each feature, taking its neighbourhood into account. Observing a feature $A_{i}$, we captured which kind of feature is at what distance to the observed one, looking at all features $A_{j}$ where j≠i. This list of labelled (by feature type) distances and the type of the observed feature itself now represent the encoding used to compare features and measure their dissimilarity.

**Figure 2:**
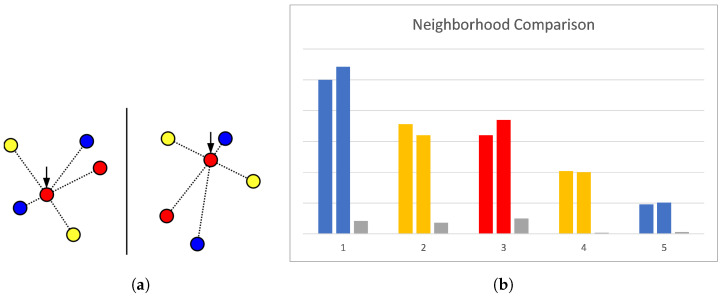
Visualization of neighbourhood comparison. Distances are measured for fixed features in each pharmacophore (**a**) and compared to compute the deviation between corresponding pairs (**b**). Colours = labels, coloured bars = encoded distances, gray bars = difference between distances.

Comparing these encodings for all features of one pharmacophore (rows) with all features of the other (columns) will then result in the values of the matrix (see Figure 2 for an example). If two encodings are representing features of different types, then the pair is marked invalid, otherwise they are compared by solving a mathematical assignment problem for which the algorithm of Jonker and Volgenant [30] is used. There, the difference is computed for each pair of encoded distances, and entries with different labels are treated as being maximally different. As too-different distances indicate that the features do not match, and starting at some difference does not have discriminative value, this difference has to be limited. For this reason, the distance difference is divided by the larger sum of positional tolerances of the two compared feature pairs. If the resulting number is larger than 1, we know that the pairs are incompatible, and the value is set to exactly 1. The total cost resulting from this assignment is then normalized by the number of features in the larger pharmacophore and represents how dissimilar the two encoded features are regarding their surroundings. This feature dissimilarity measure is used in the next step as a pseudo-cost for the assignment of one feature to the other, allowing us to fill the cost matrix.

**Figure 3:**
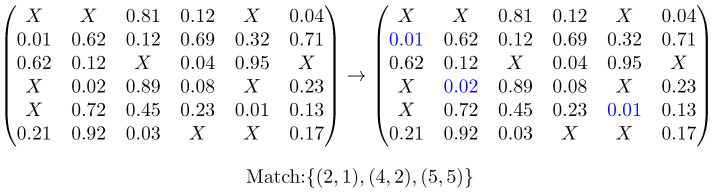
Example matrix used for the assignment of matching pairs. X indicates feature mismatches, marking a given pairing as impossible.

From this matrix, we can obtain a guess by solving another mathematical assignment problem and picking the three matches with minimal dissimilarity values. An example of this can be seen in Figure 3.

This guess should in theory represent the best option for running an iterative refinement process. Still, as we abstracted the 3D information of where points are located relative to each other to simple distances, our guess may not always result in the optimal solution after refinement. This could, for example, be the case if the pharmacophores would only fit after a symmetry transformation. To combat these non-optimal guesses, we propose methods to obtain multiple starting combinations.

**Multiple Guesses.** To obtain an arbitrary number of guesses *m*, we can reuse the dissimilarity matrix mentioned above. Iterating over this matrix, we can efficiently check the feasibility of every combination of three pairs, keeping track of the *m* best ones. To compute how good a guess is we simply sum up the dissimilarity values for the three pairs, with a smaller sum indicating a better choice. Note that even though this process has an algorithmic complexity of $O(n^{6})$ (where *n* is the number of pharmacophore features), this is in practice very fast, as permutations of the same three pairs can be ignored, and pairs with incompatible feature types can be skipped. We also found that this guess-generating process is negligible in runtime compared to the iterative refinement of the found guesses.

**Trying All Possibilities.** It is also possible to just use all combinations of three pairs as starting points for iterative refinement. For this, we do not need to compute a dissimilarity matrix for features but just keep track that all pairs contain features of the same type. This should result in the most accurate alignment, as the largest possible number of starting combinations is sampled, but it has the downside of being more computationally expensive, due to the time needed for refinement. Still, this should be faster than choosing a different algorithm altogether as there are fewer three-pair combinations than if one computes all possible combinations (of all sizes) for the features, as other methods may do.

#### 2.2.2. Collecting Additional Pairs

For each initial guess of three matching pairs, iterative refinement is performed to both align the collected pairs and to find additional pairs that fit. This idea is similar to the Iterative Closest Point (ICP) algorithm [31], which is usually used to register unordered point clouds from rigid objects by iteratively matching points, evaluating the quality of the transformation and improving on it. Our implementation was modified to better represent the goal of maximizing the number of features with overlapping threshold spheres, while having the same feature type. The concrete steps of the method are as follows:

- Given a list of starting matches *P* (three initial pairs),
- Align pairs *P* using the Kabsch Algorithm.
- Build a matrix *F* “forbidden” for all feature pairs.
  – Mark pairs with different types forbidden.
  – Mark columns and rows related to features in P forbidden.
- While *F* contains unmarked entries:
  – Iteratively improve *P*.
- Return matching pairs and alignment matrix.

The matrix *F* tracks which pairs could be feasible to expand the matched feature pairs and can indicate when the algorithm should be terminated. Each iteration, this improvement process checks a possible addition to the matching pairs and computes a new alignment while setting at least one entry of *F* as forbidden. Due to the strict increase in marked entries, one can be sure that the method terminates. Additionally, one can stop this process earlier if all features match or if we only care about finding any alignment, while omitted features are allowed and have a sufficiently large matching. Each iteration of the improvement performs the following steps:

- Find closest pair (by distance) $p_{test}$ not marked in *F*.
- Create temporary pair combination $P_{test}=P\cup p_{test}$.
- Align pairs $P_{test}$.
- If $P_{test}$ does not fulfill threshold,
  – Try applying translation.
- If $P_{test}$ does still not fulfill threshold,
  – Delete $P_{test}$.
  – Mark pair $p_{test}$ as forbidden in *F*.
  – Restore alignment of *P*.
  – Continue.
- Otherwise,
  – Set $P=P_{test}$.
  – Mark rows and columns related to $p_{test}$ in *F* forbidden.
  – Continue.

In many cases, it is not necessary to compute the alignment for all of the guesses presented. After refining a guess, the contained tree-pair combinations are stored, and subsequent guesses, starting with three points that were already collected by a previous refinement, are skipped. This is done as in most cases, and refining from those pairs would converge toward the same solution as was previously found.

#### 2.2.3. Post-Processing

In the post-processing step, additional requirements for a valid alignment are checked. One of these checks is the collision with exclusion volumes (see Section 2.1), for which adjustments can sometimes be made. Other than that, features may come with more specialized requirements, for which we can only decide if they are met or not and thereby keeping or discarding the solution. These restrictions on the results are especially useful in screening, as reducing the number of results by using domain knowledge is beneficial for further processing.

**Figure 4:**
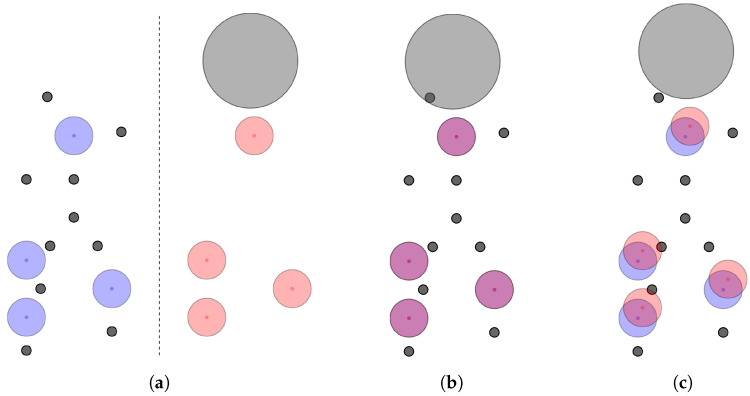
Illustration of exclusion volume clashes leading to an invalid alignment. For the sake of simplicity, pharmacophores (**a**) consist only of features of the same type and atoms of one pharmacophore, shown as black circles. After an alignment of the pharmacophores, atoms may clash with exclusion volumes (**b**), which can sometimes be fixed with a simple translation (**c**).

**Dodging Exclusion Volume Spheres.** If the resulting alignment includes atoms that collide with exclusion volumes, additional translations (currently up to three) can be applied to try to “dodge” those exclusion volumes to save the alignment. An example for this is provided in Figure 4 where an exclusion volume would lead to discarding an otherwise good alignment solution.

After those translations, if there are still collisions, the alignment has to be discarded, otherwise the pharmacophore feature thresholds for matched pairs need to be checked again. Based on whether or not the features still are within their allowed threshold distances, the alignment may now be valid or not. This may save a solution that only had a minor exclusion volume clash from being discarded even though it may have been a reasonable alignment.

#### 2.2.4. Other Requirements

Other requirements imposed by pharmacophore features could include a variety of properties like direction vectors or plane normals. Here, as part of the model, one can assume the direction in which the feature should point, and any matching features should also roughly point in that direction after alignment. If this is not the case, the feature is considered unmatched,, and if this leads to less-matched features than required, the solution is discarded. This could also be an interesting topic for further investigation in a follow-up work, as it may be possible to consider these direction vectors in the alignment process itself.

## 3. Results and Discussion

### 3.1. Benchmarking Methodology

To benchmark the number of found matches and the required computation time, we implemented our alignment algorithm in a development version of LigandScout for use in virtual screening. Here, the goal was to find molecules, abstracted by pharmacophores, matching one or more query pharmacophores. Since also a significant amount of completely unfeasible pharmacophore combinations would be aligned in a typical virtual screening run, pre-processing filters are usually applied to reduce the overall computation time [8]. This was not done in our benchmarks as we only cared to measure the time spent for the actual alignment process.

To obtain more sensible results, the hits were again filtered after the alignment. Here, alignments were discarded if the features did not meet additional requirements like the directions of feature vectors not being sufficiently close. If there were still clashes with exclusion volumes (after trying to fix those), the alignment was also discarded. The remainder of the found solutions was then accepted and presented as the output hit-list. This filtering based on model restrictions was performed equally for both of the benchmarked algorithms.

Additionally, note that the pharmacophores benchmarked were not manually optimized for separating actives from inactives/decoys. Even though this is what one wants to achieve in practice, the type of retrieved molecules should only depend on the quality of the model. In constrast, the alignment method (which was the focus of our benchmarks) should be able to accurately retrieve all molecules that fit the query model. For this reason, finding additional hits, regardless of if they are actives or decoys, indicates that the method that did not find them was not able to fulfil the user query accurately and omitted results the user asked for. As a side effect of using a more-accurate alignment method and retrieving molecules that fit the query model more precisely, one may need to adjust the model as, in expectation, more hits will be found. As this is often not desirable, reducing the size of feature spheres or additionally filtering the hits with stronger restrictions after screening may be advisable.

All computations for the measurements below were run multi-threaded on an AMD Ryzen 9 5900X 12-Core (24 threads) 3.7 Ghz CPU in Windows 10, taking the average of five runs. Before the first measured run, one full non-measured benchmarking run was performed as a “warmup phase” to ensure the different methods and runs neither benefited nor got punished for start-up-related factors like the CPUs branch prediction and I/O caching.

### 3.2. Results

First, we assessed the impact of the chosen number of computed guesses for our G3PS algorithm. For this, we compared different datasets in regard to the computation time needed per guess and the number of hits found depending on the number of allowed omitted features.

**Figure 5:**
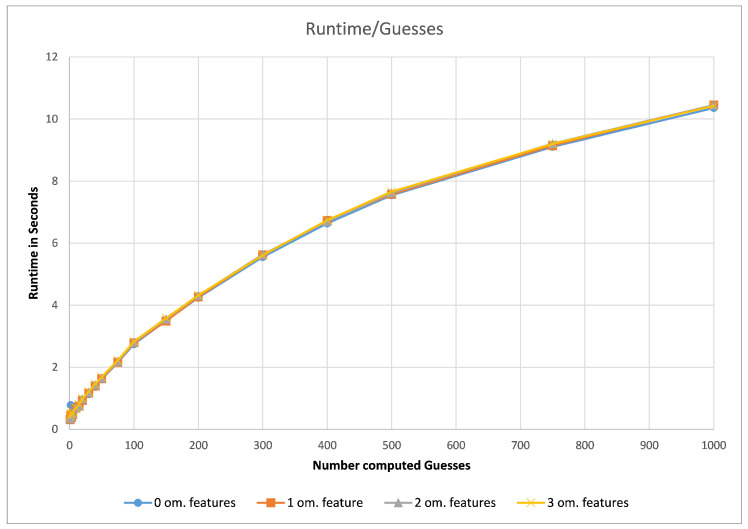
CDK2, runtime (full dataset) vs. computed guesses.

As can be seen in Figure 5 (using the CDK2 dataset as an example), the runtime of our method did not depend on how many omitted features are allowed but purely the number of computed guesses. As the number of guesses increases, the iterative improvement will check combinations of features that it has seen before. Therefore, the increase in runtime is less than linear in the number of guesses. To now find a good default value, we analysed the number of hits with different parameter values.

**Figure 6:**
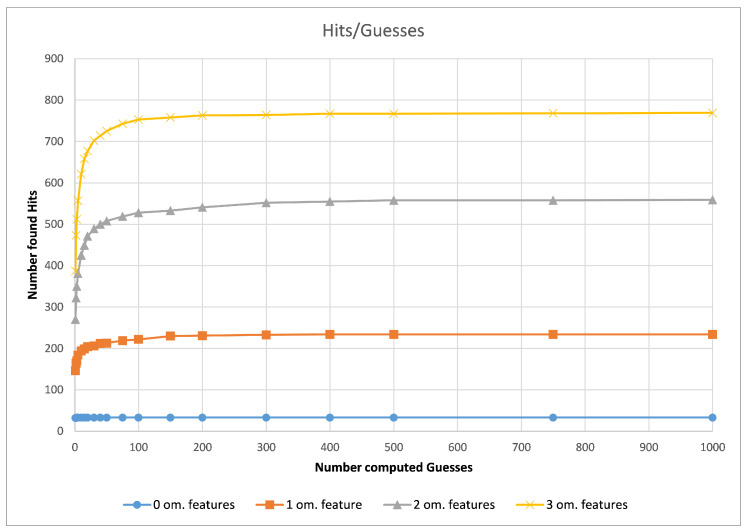
CDK2, number of hits vs. computed guesses.

Figure 6 shows that, in practice, only a few guesses are needed to find the majority of hits. In our experiments, we observed that 20 guesses lead to fast and accurate results, while 300 guesses found the maximum amount of hits most of the time. In the following, we call the 20-guess variant “G3PS Fast” and the 300-guess variant “G3PS Accurate”.

**Figure 7:**
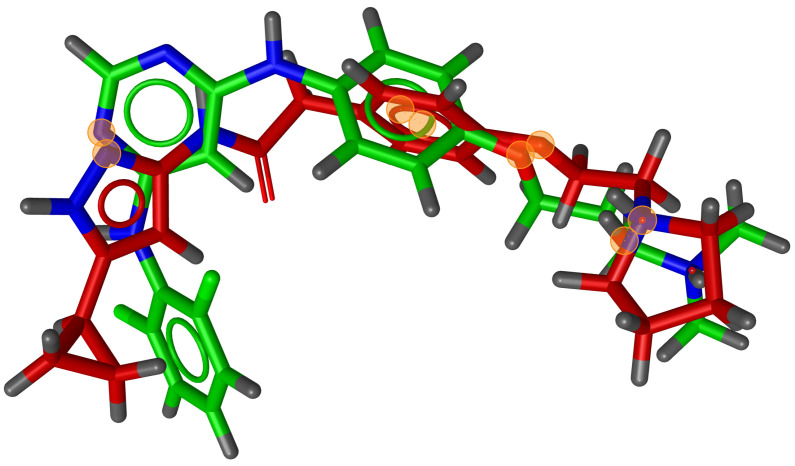
RM method example alignment of two molecules; four matching feature pairs were found. Orange circles = centre of matched features.

**Figure 8:**
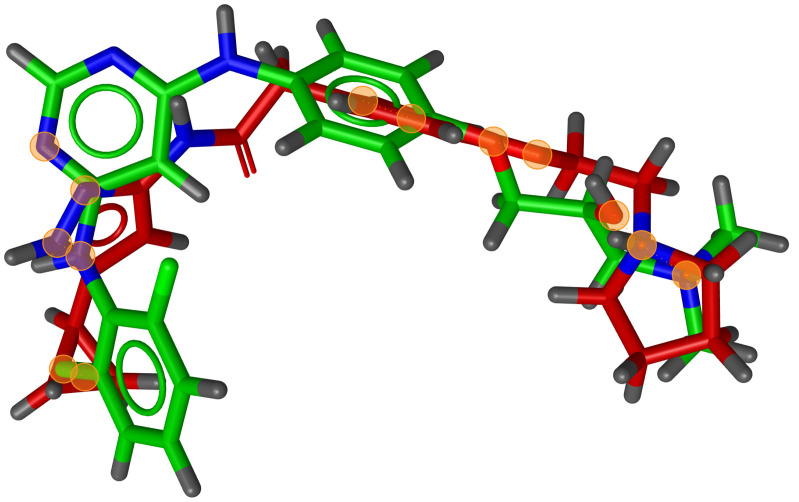
G3PS example alignment of two molecules; seven matching feature pairs were found. Orange circles = centre of matched features.

During testing, we visually analysed differences between RM and G3PS. An example can be seen in Figure 7 and Figure 8, where the same two molecules were aligned based on their pharmacophore features with both methods. The RM method found four matching feature pairs and aligned the molecules according to those while G3PS (accurate) found and aligned by seven matching feature pairs.

**Figure 9:**
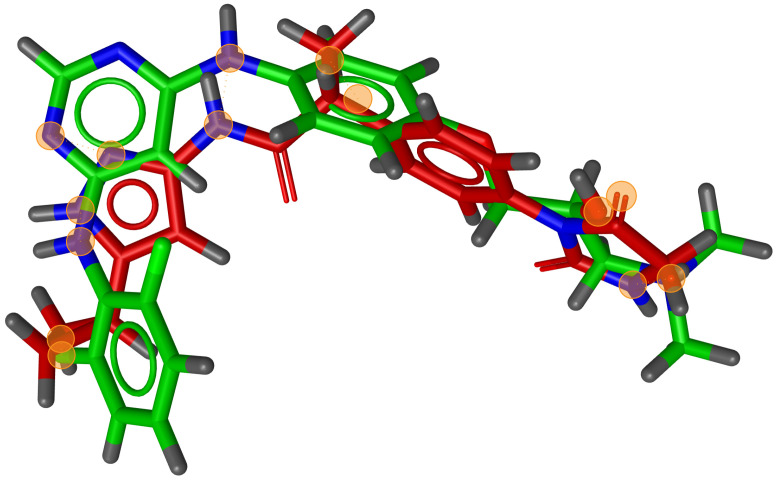
Example of G3PS alignment with seven matching feature pairs where RMM failed due to not finding three pairs. Orange circles = centre of matched features.

We also found that many combinations of molecules could not be aligned with the RM method as it could not identify three matching feature pairs. An example of such a combination can be seen in Figure 9, where seven matching feature pairs were identified by G3PS (accurate) and used for alignment.

For the benchmarking datasets, we selected 10 different targets from the DUD-E database [28,29] and just used the known active ligand sets. For these molecules, up to 25 conformations were created using LigandScout’s iCon conformer generator [11]. For use as query pharmacophores, structure-based models were created using DUD-E’s provided receptor and co-crystallized ligand structure, removing excess features until hits could be found. The exclusion volumes that were created in the modelling process were kept. These pharmacophores were not further optimized for practical use and only served for benchmarking the accuracy and performance of the methods, disregarding the selectivity of actives vs. decoys (see Section 3.1). Images of the pharmacophores can be found in our Supplementary Materials. The used DUD-E datasets and the number of pharmacophore features (after removing excess ones) are shown in Table 1.

Note that we included the GCR dataset and used a pharmacophore with nine features to also measure the performance and accuracy with comparatively larger models. Due to this large number of features, the model was more selective than the ones from the other datasets, and fewer found hits were expected regardless of the chosen alignment method.

In the following, all comparisons are made to the RM algorithm [15]. Both methods use the same file back-end and processing pipeline.

**Figure 10:**
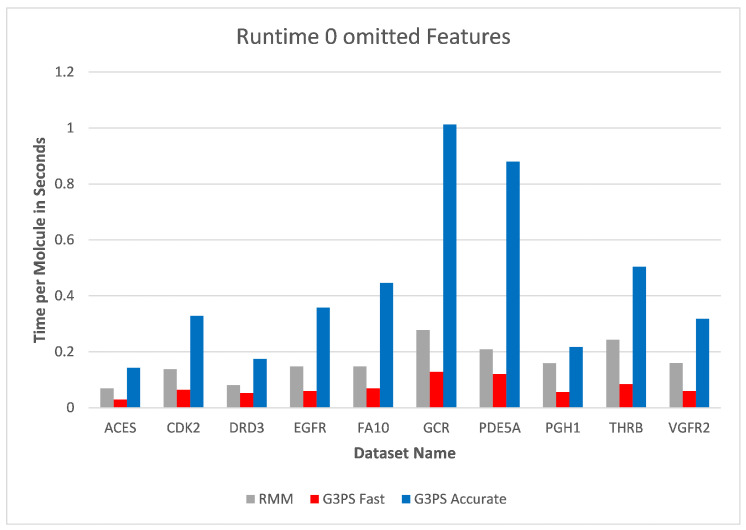
Screening benchmark runtimes without omitted features.

**Figure 11:**
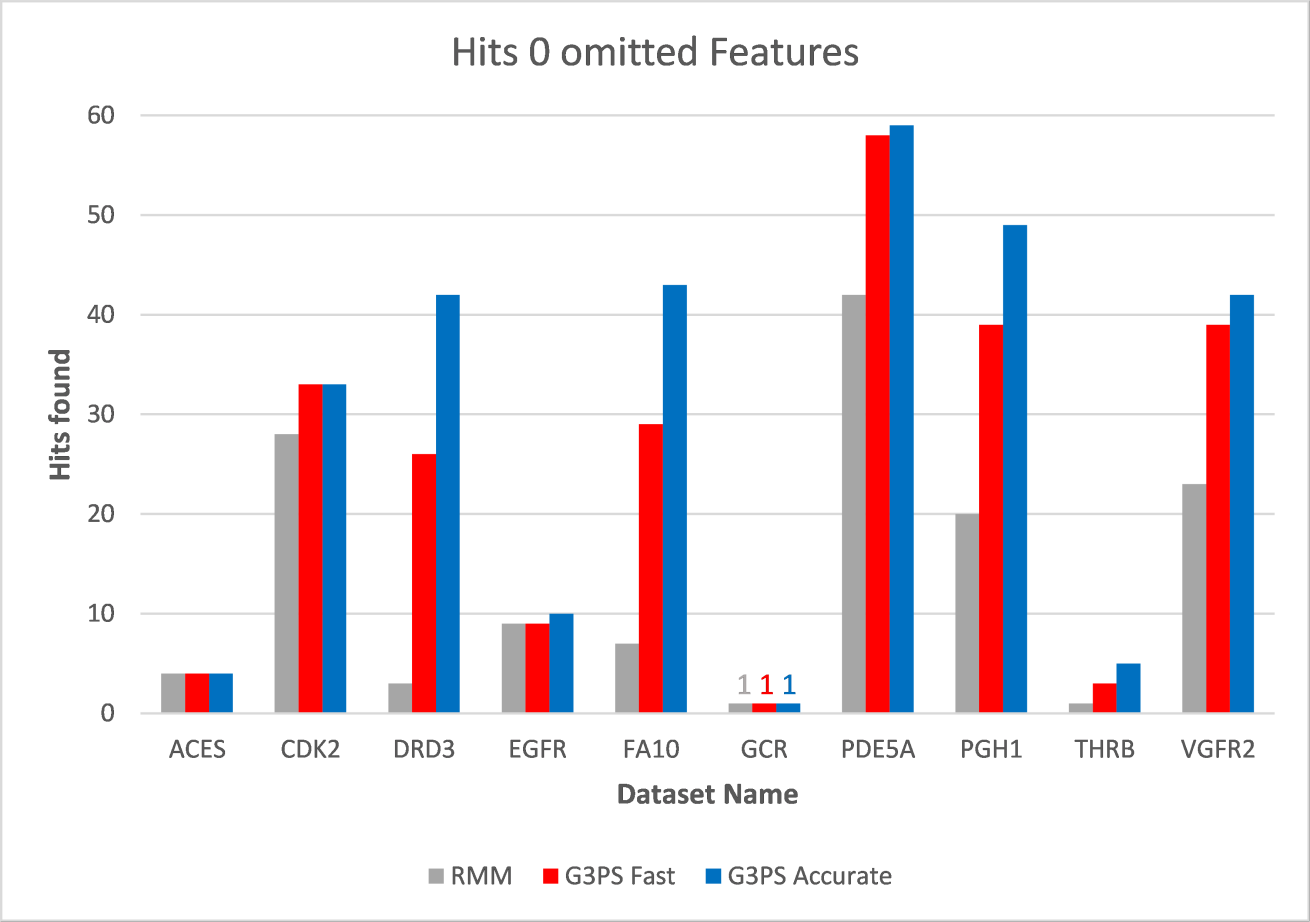
Screening benchmark hits without omitted features.

The runtime and resulting hits for our virtual screening experiments without omitted features can be seen in Figure 10 and Figure 11. Our G3PS “Fast” variant was able to retrieve hits more accurately than the RM method while still being faster. For the measured datasets, this is a speed-up (time RMM/time G3PS Fast) of approximately 2.3× on average. Using the “Accurate” variant settings, one can sometimes find additional hits at the cost of a longer runtime. This may be a valid option for users that do not want to miss hits and that are willing to accept longer computation times.

**Figure 12:**
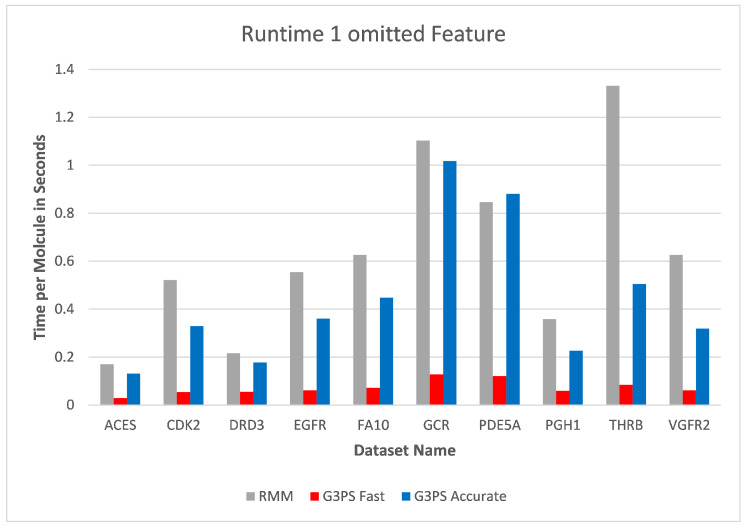
Screening benchmark runtimes with one omitted feature.

**Figure 13:**
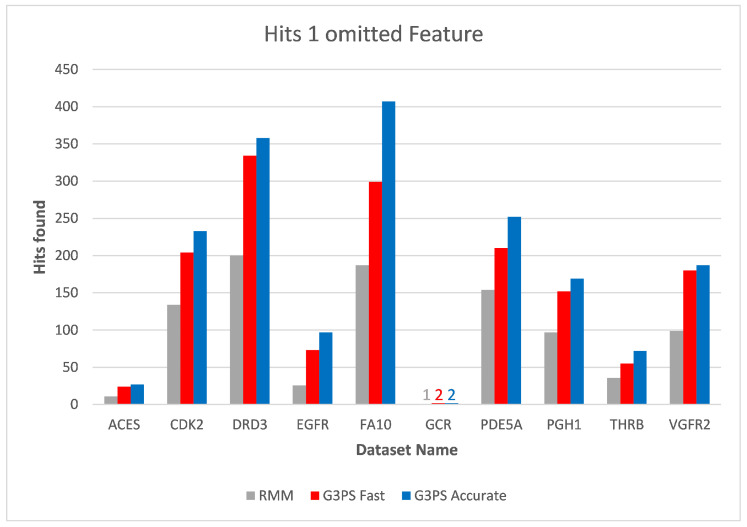
Screening benchmark hits with one omitted feature.

Starting at one omitted feature (Figure 12 and Figure 13), the runtime of screening with the RM method increased with the number of omitted features, while staying constant with G3PS. Our “Fast” method still provided comparatively more hits while extending its runtime advantage (on average, an 8.5× speed-up). Even our “Accurate” variant was competitive in runtime, only being slightly slower for the PDE5A dataset. It still computed an even larger amount of hits for all datasets, while now being faster than the reference method for all other datasets.

**Figure 14:**
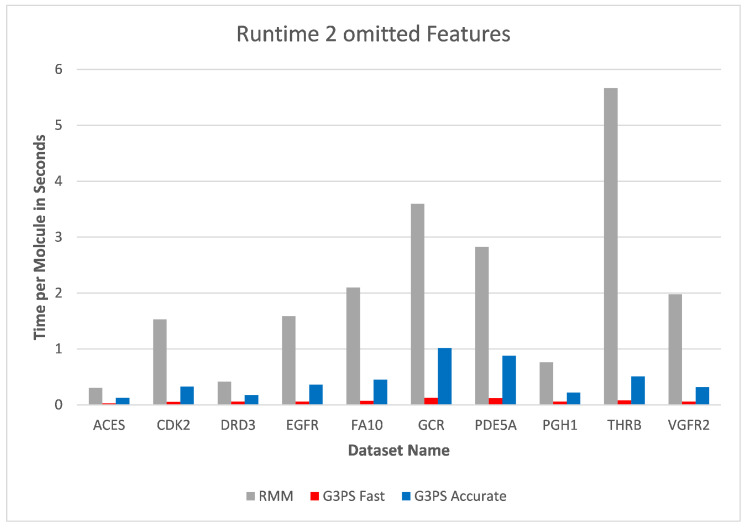
Screening benchmark runtimes with two omitted features.

**Figure 15:**
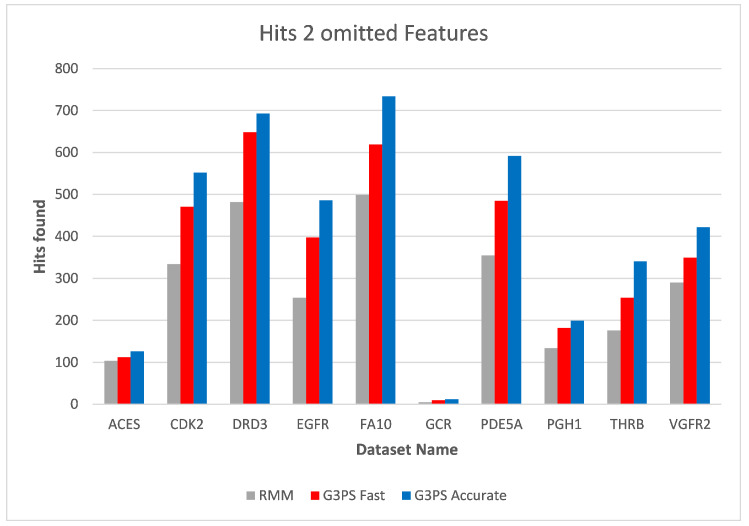
Screening benchmark hits with two omitted features.

Increasing the number of omitted features to two (Figure 14 and Figure 15) again increased the runtime of the RM algorithm. At this point, both presented presets for G3PS performed better in regard to runtime and the number of found hits. G3PS “Accurate” was now on average 4.6 times faster, and the “Fast” variant was 25.8 times faster.

**Figure 16:**
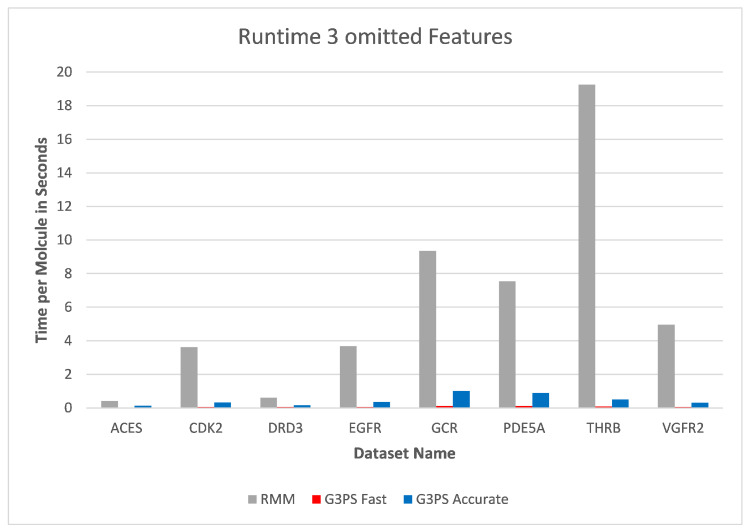
Screening benchmark runtimes with three omitted features.

**Figure 17:**
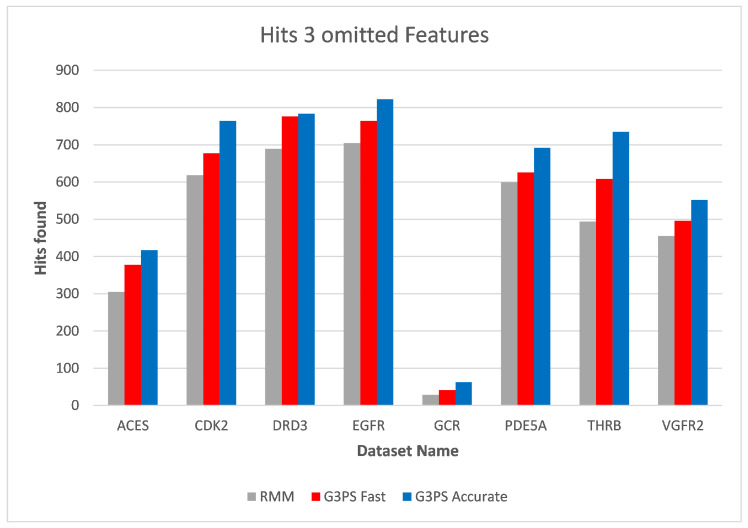
Screening benchmark hits with three omitted features.

As already seen with two omitted features, three omitted features (Figure 16 and Figure 17) continued to show the independence of screening with G3PS to this number. At this point, the RM method starts becoming unfeasible for practical use, like for fragment screening where a larger number of omitted features is necessary. Even though the runtime increases this drastically, it is again not able to retrieve hits as accurately as our “Fast” variant. Our speed-up further increased to 70.7× and 12.3× for our “Fast” and “Accurate” variants, respectively. Two datasets could not be benchmarked with three omitted features, as the pharmacophores only contained five features, and removing three of those would result in a pharmacophore for which a defined, unambiguous 3D alignment is not possible.

### 3.3. Summary

The Greedy 3-Point Search method for alignment brings a variety of benefits over existing methods. With translations applied in the iterative refinement and the general method of collecting additional pairs based on three starting pairs, our algorithm was able to optimize for maximum matching feature counts. In this way, alignments are not missed that should actually be possible for the two given pharmacophore models. As the algorithm looks for the largest possible match iteratively instead of computing alignments for combinatorially generated feature subsets, the number of omitted features does not impact the runtime, making it orders of magnitude faster than competing methods. With this iterative refinement process, the problem that more omitted features could lead to more found hits than with fewer omitted features due to feature centroid changes is also avoided. The independence from the omitted feature count makes our method especially suitable for fragment screening where a larger model needs to be aligned to multiple very small fragment pharmacophores, resulting in a high number of required omitted features. The algorithm can also be tuned by the user for accuracy or performance by adapting the number of sampled refinement starting points, while a small number of starting points still finds the best alignment most of the time. Another commonly found weakness of existing methods is that alignments may be discarded because exclusion volumes are slightly touched, even though very minor changes would allow for a valid, possibly even near-optimal solution. G3PS can avoid this problem in the post-processing step by applying minor translations, thereby not dropping such good alignments.

### 3.4. Future Directions

A logical extension to the iterative refinement process could be to include additional information like feature-direction vectors already in this stage. This would allow for an earlier detection of feature pairings where not all constraints can be met and thus could potentially achieve an improvement in runtime. Alternatively, one could try to consider constraints like feature directions during the alignment itself. This could improve the positioning in space such that these restrictions are more likely to be fulfilled without additional intervention and could thereby reduce the number of guesses needed to find a good alignment.

Further research could also be targeted at extracting parts of the method for GPU computation. A large part of our algorithm consists of fundamental mathematical operations, and additional performance can possibly be gained by using the computational resources provided by graphics accelerators.

## 4. Conclusions

We presented a new algorithm, Greedy 3-Point Search, for aligning two pharmacophores in a way that the number of matching features is maximized. This avoids the problem of missing valid alignments due to an unfit optimization target, resulting in a higher number of solutions in agreement with the specified positional tolerances of the pharmacophoric features. The new method iteratively refines starting guesses, finding the largest match without having to consider the allowed number of omitted features. For this reason, the runtime of the algorithm does not depend on omitted features but only the number of starting guesses used. This allows a user to find a trade-off between high accuracy of results and low execution time, while still obtaining good results when the fast variant is chosen. For ease of use, we proposed two default parameter settings for the number of starting guesses, which should cover the needs of most users. Additionally, we touched on exclusion volume restrictions and how to recover from alignments that were deemed invalid due to only barely violating exclusion volume constraints.

As our method is able to find hits more accurately, and thereby presents the user more screening hits overall, it will be important to look into how this affects the way pharmacophores are usually refined by expert users. It may now be of benefit to reduce positional tolerances or to narrow down allowed angle deviations for directed features to improve a pharmacophore model’s ability to discriminate between active and inactive molecules. Aside from its impact on virtual screening results, the higher probability to find valid alignments will also have an influence on the results obtained by other pharmacophore-based techniques. For lead optimization tasks, the robustness of our algorithm will significantly extend the possibilities when it comes to the structural modification of molecules. Pharmacophore-guided replacements of chemical functionalities by bioisosteric groups or even scaffold hops have a higher chance to succeed due to the largely reduced risk of failures when attempting a realignment of the template pharmacophore. For ligand-based pharmacophore modeling procedures, following a feature-count maximizing alignment strategy will, on average, result in the perception of a higher amount of common training-set ligand features than one would obtain using a pure RMSD-minimizing strategy. Unfortunately, the average impact of our new alignment algorithm on the outcome of these pharmacophore-based techniques cannot be quantified easily since several other methods and case-specific factors are also of influence and need to be accounted for. However, we are confident that taking advantage of the robustness and the feature-count maximizing search strategy of our new algorithm would be beneficial for any pharmacophore-based technique relying on pharmacophore alignment and thus would contribute to its further development.

## Figures and Tables

**Table 1:** Summary of benchmarking datasets.

Name	Pha. Features	Molecules	Conformations
ACES	6	664	15,391
CDK2	6	798	17,740
DRD3	6	877	20,539
EGFR	6	832	19,899
FA10	5	792	19,786
GCR	9	563	9610
PDE5A	7	706	17,015
PGH1	5	251	4041
THRB	7	861	21,474
VGFR2	6	620	15,041

## Supplementary Materials

The following are available online. Images of pharmacophores used for benchmarking: ACES, CDK2, DRD3, EGFR, FA10, GCR, PDE5A, PGH1, THRB, VGFR2.
