# Supplementary figures and images for: Greedy 3-Point Search (G3PS)—A Novel Algorithm for Pharmacophore Alignment
Source: Molecules. 2021 Nov 27;26(23):7201. doi: 10.3390/molecules26237201 (PMC8658842; doi:10.3390/molecules26237201)

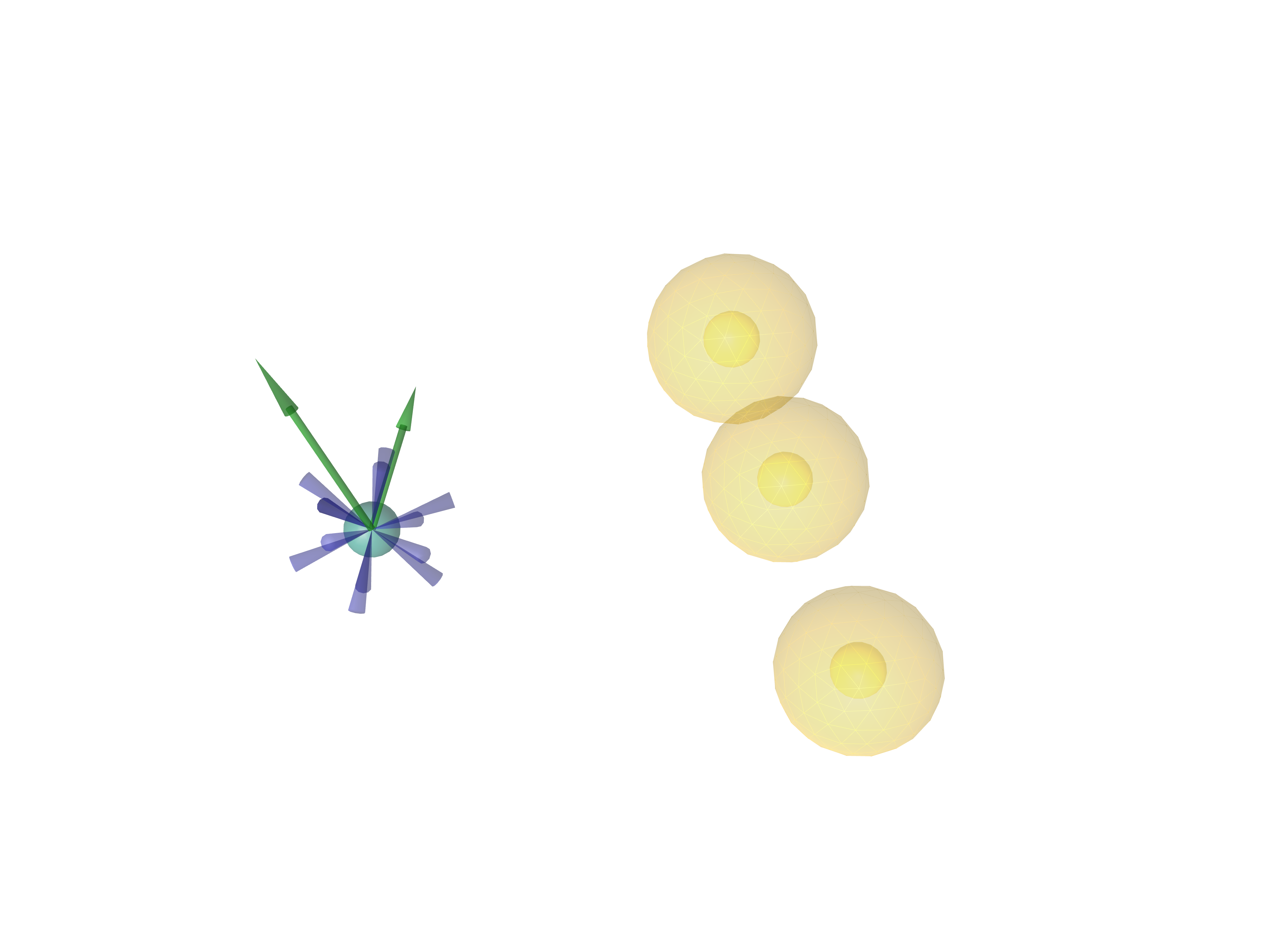

Supplement: Supplementary file 1 [file molecules-26-07201-s001.zip › pha/DRD3.png]

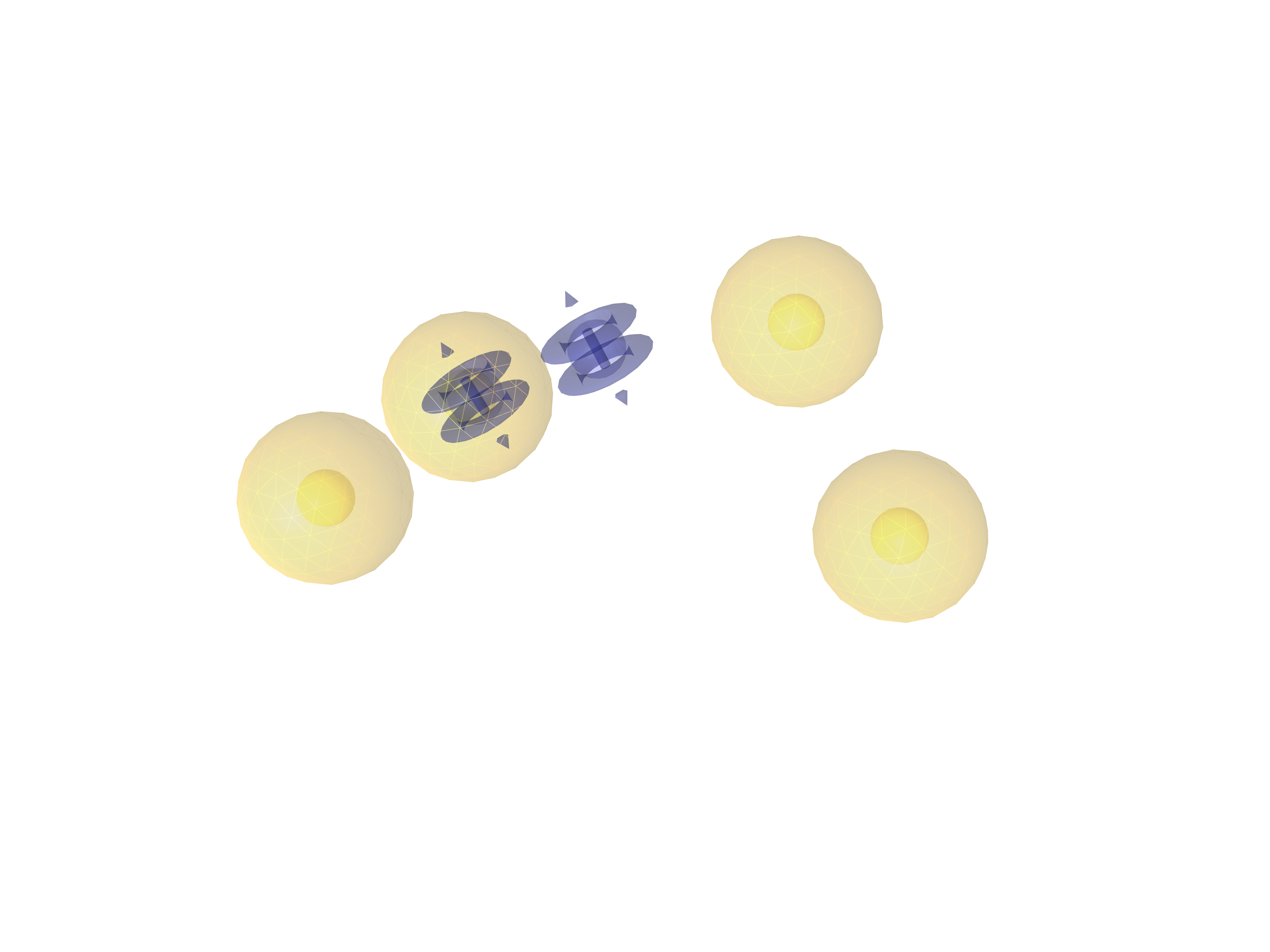

Supplement: Supplementary file 1 [file molecules-26-07201-s001.zip › pha/ACES.png]

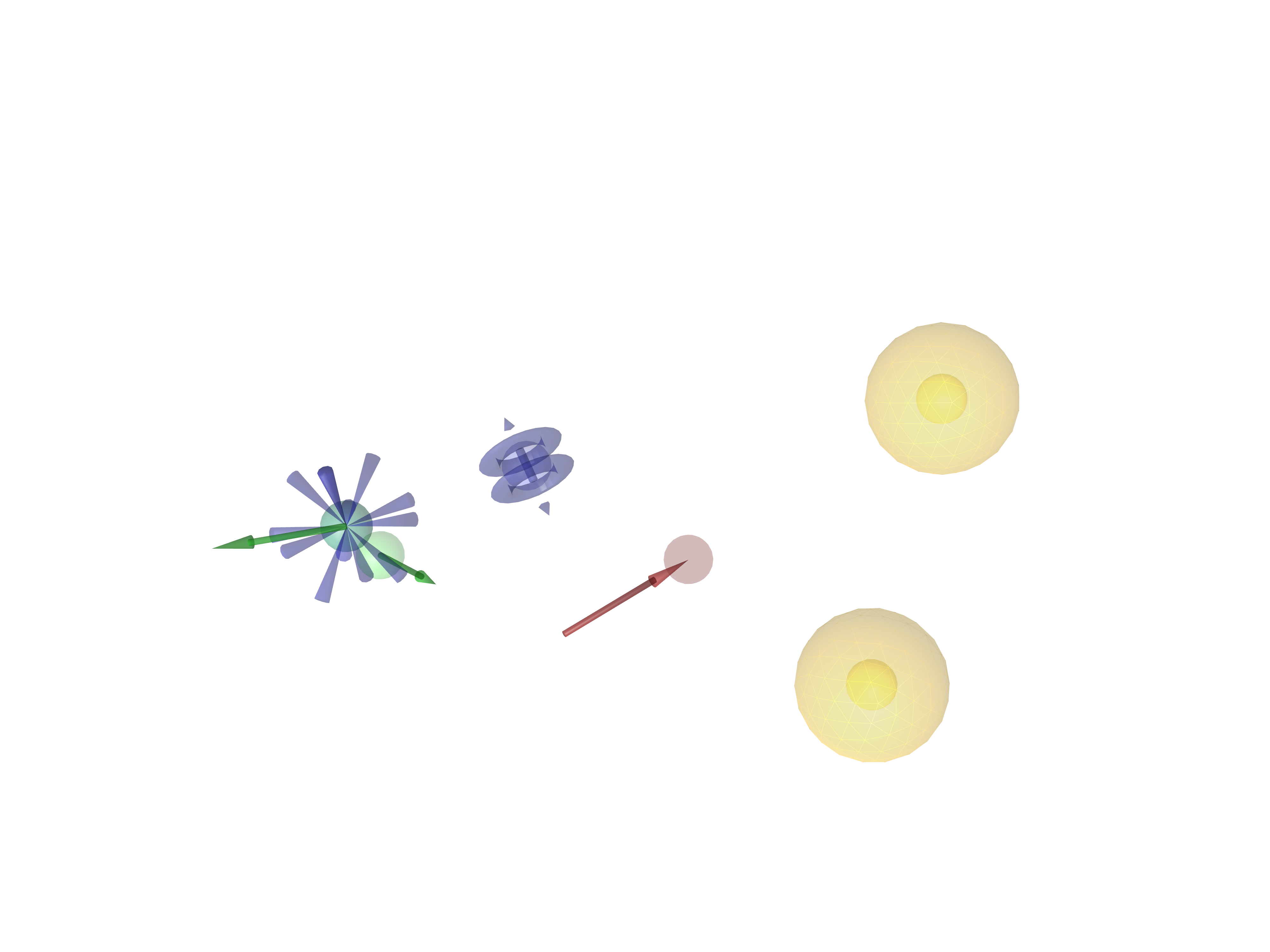

Supplement: Supplementary file 1 [file molecules-26-07201-s001.zip › pha/THRB.png]

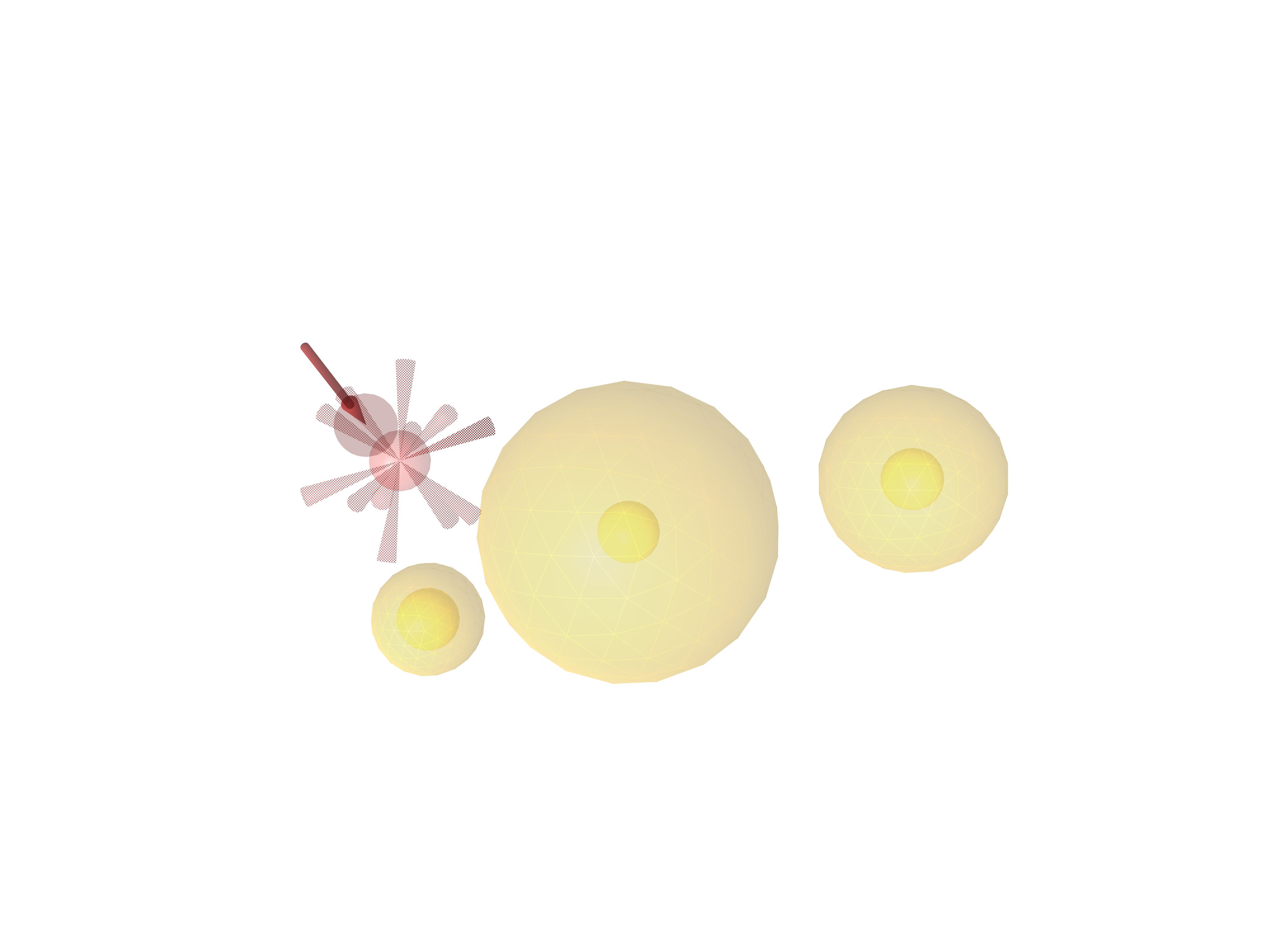

Supplement: Supplementary file 1 [file molecules-26-07201-s001.zip › pha/PGH1.png]

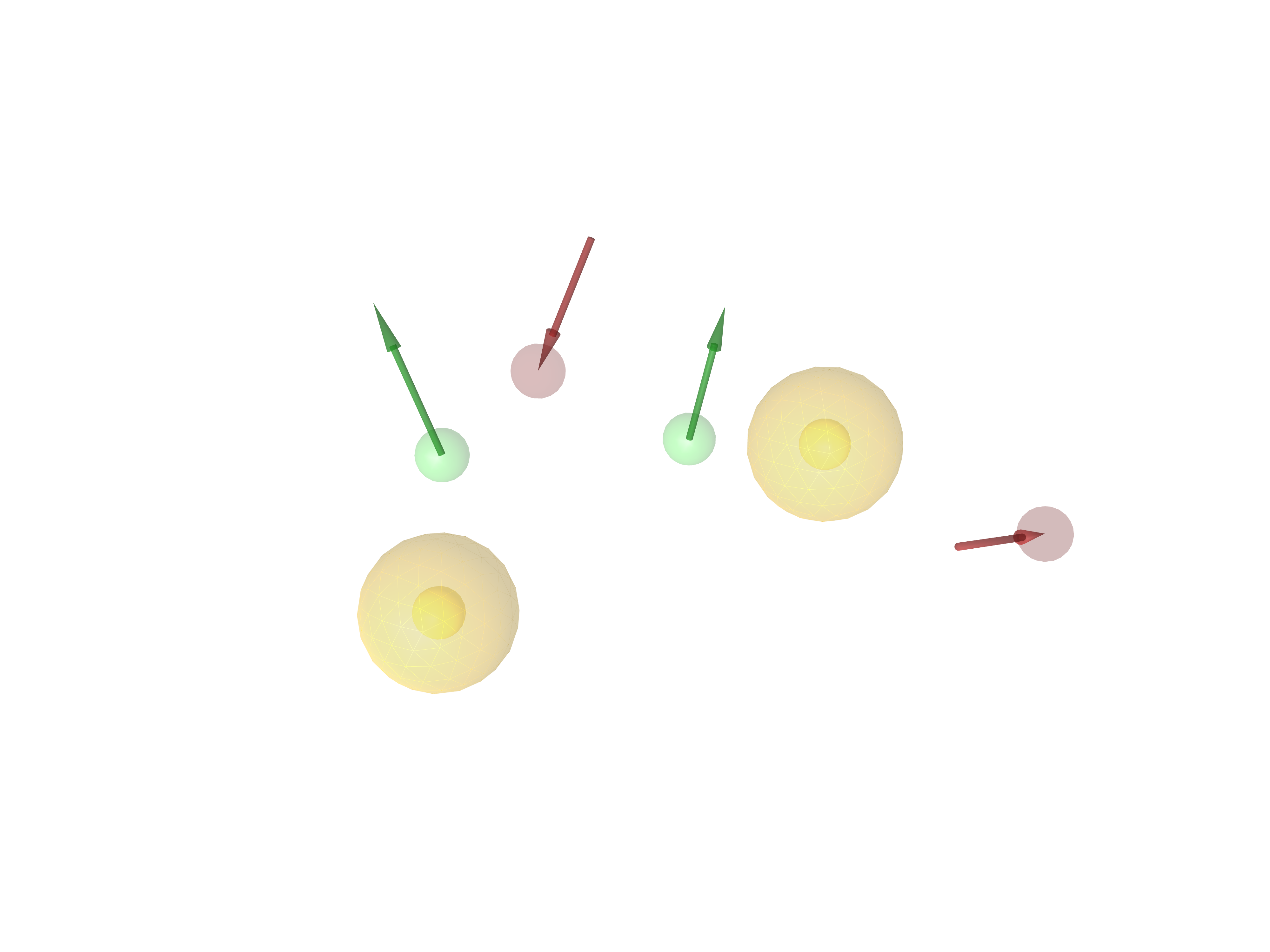

Supplement: Supplementary file 1 [file molecules-26-07201-s001.zip › pha/CDK2.png]

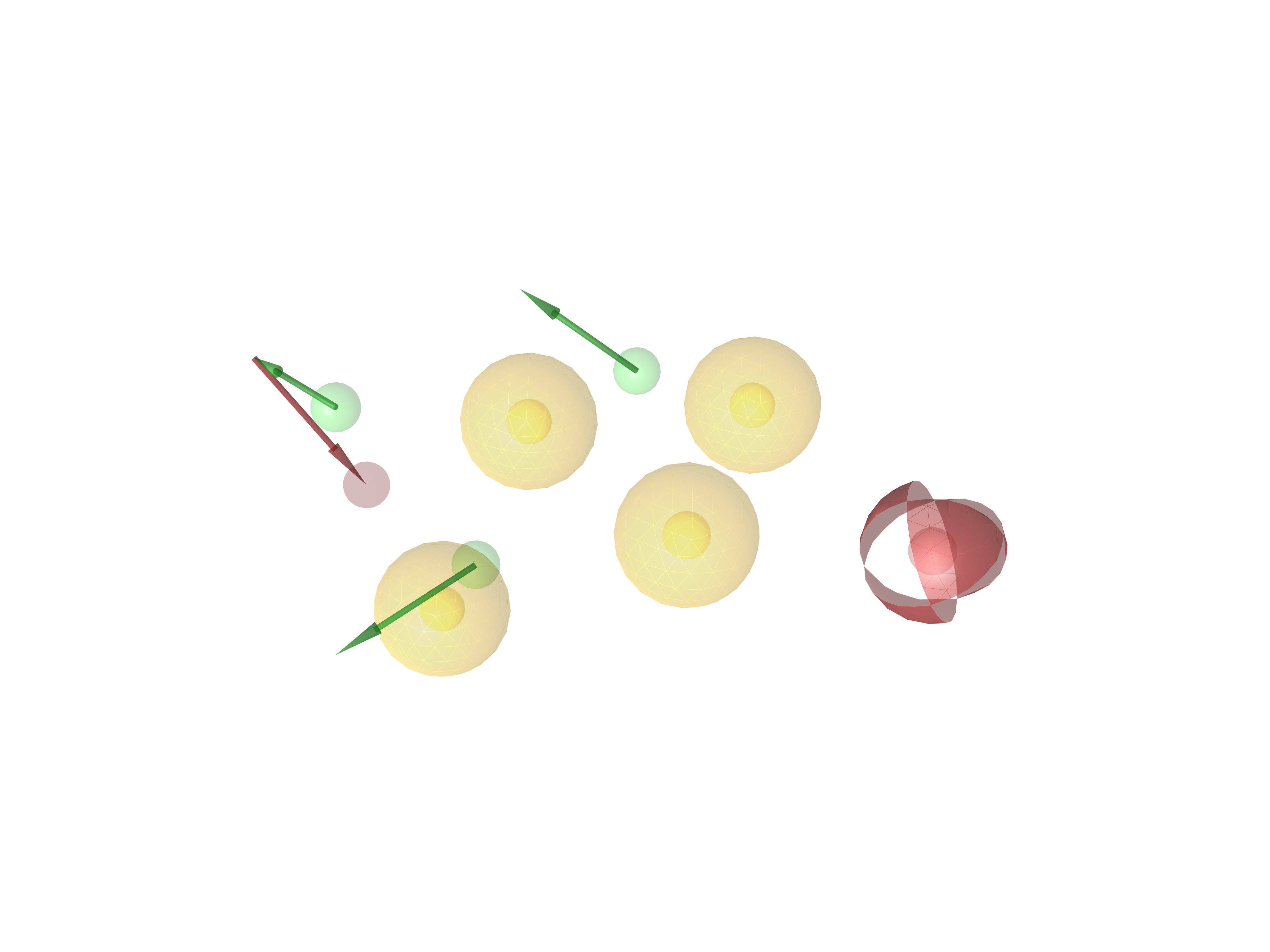

Supplement: Supplementary file 1 [file molecules-26-07201-s001.zip › pha/GCR.png]

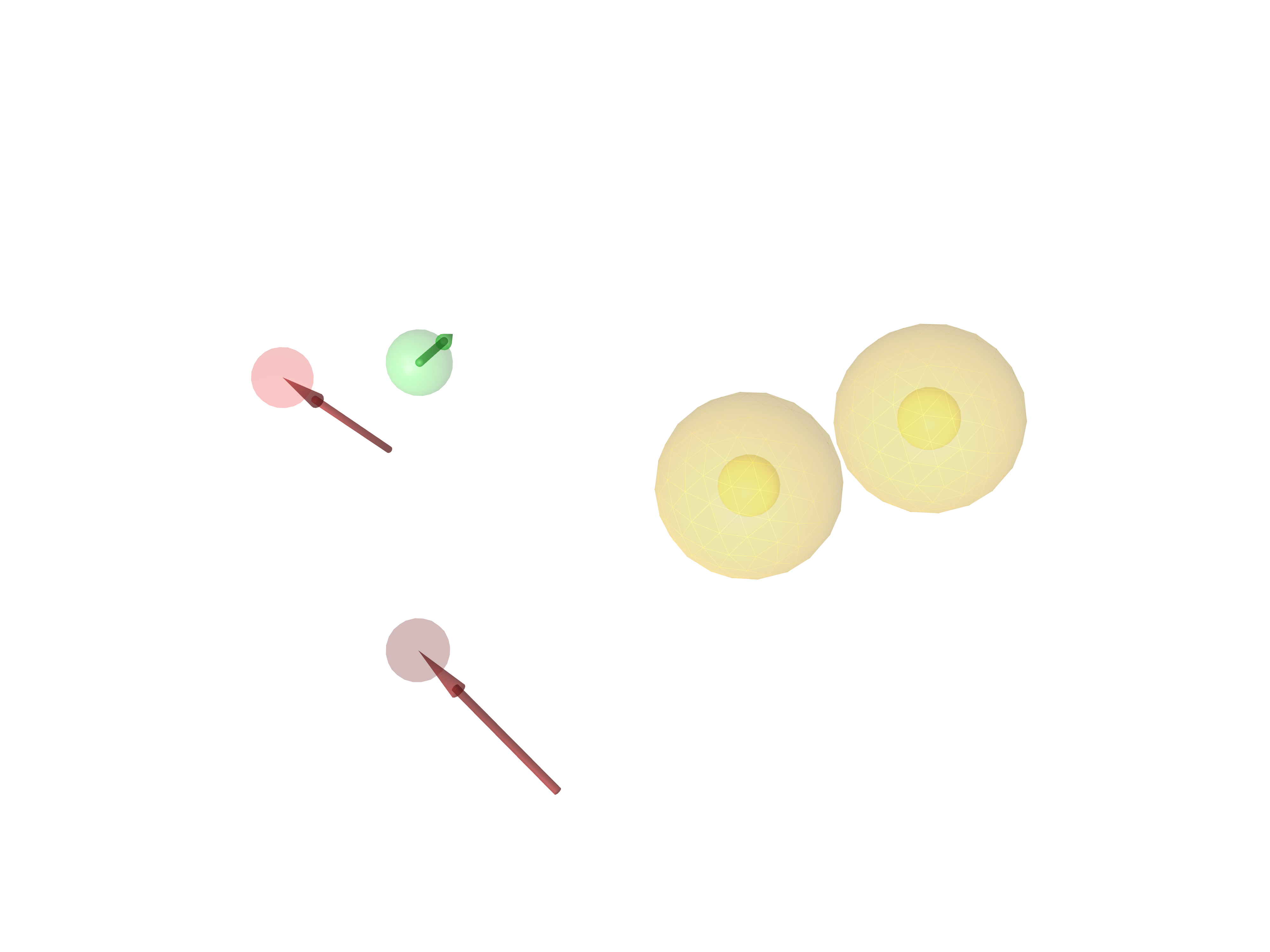

Supplement: Supplementary file 1 [file molecules-26-07201-s001.zip › pha/FA10.png]

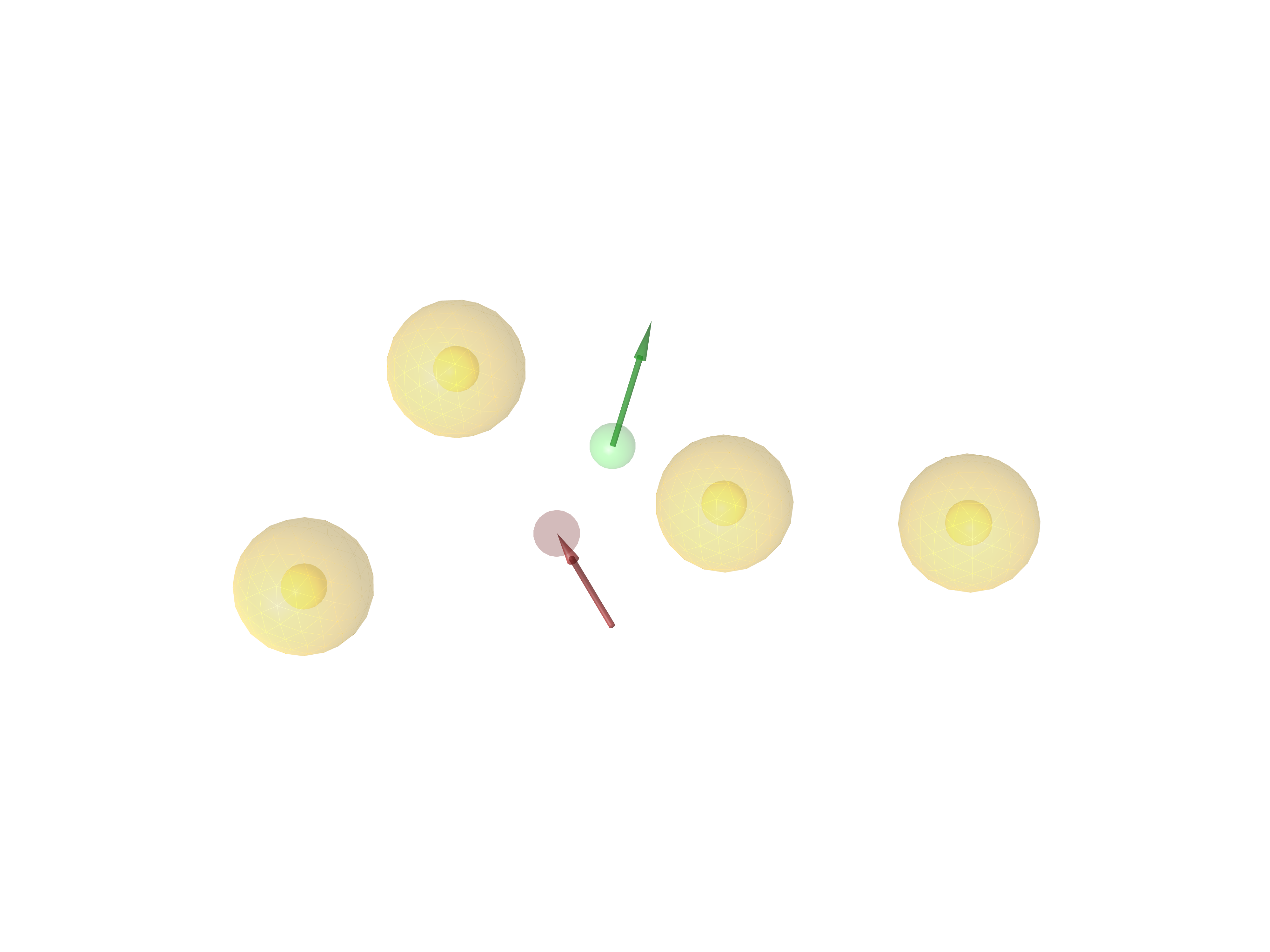

Supplement: Supplementary file 1 [file molecules-26-07201-s001.zip › pha/VGFR2.png]

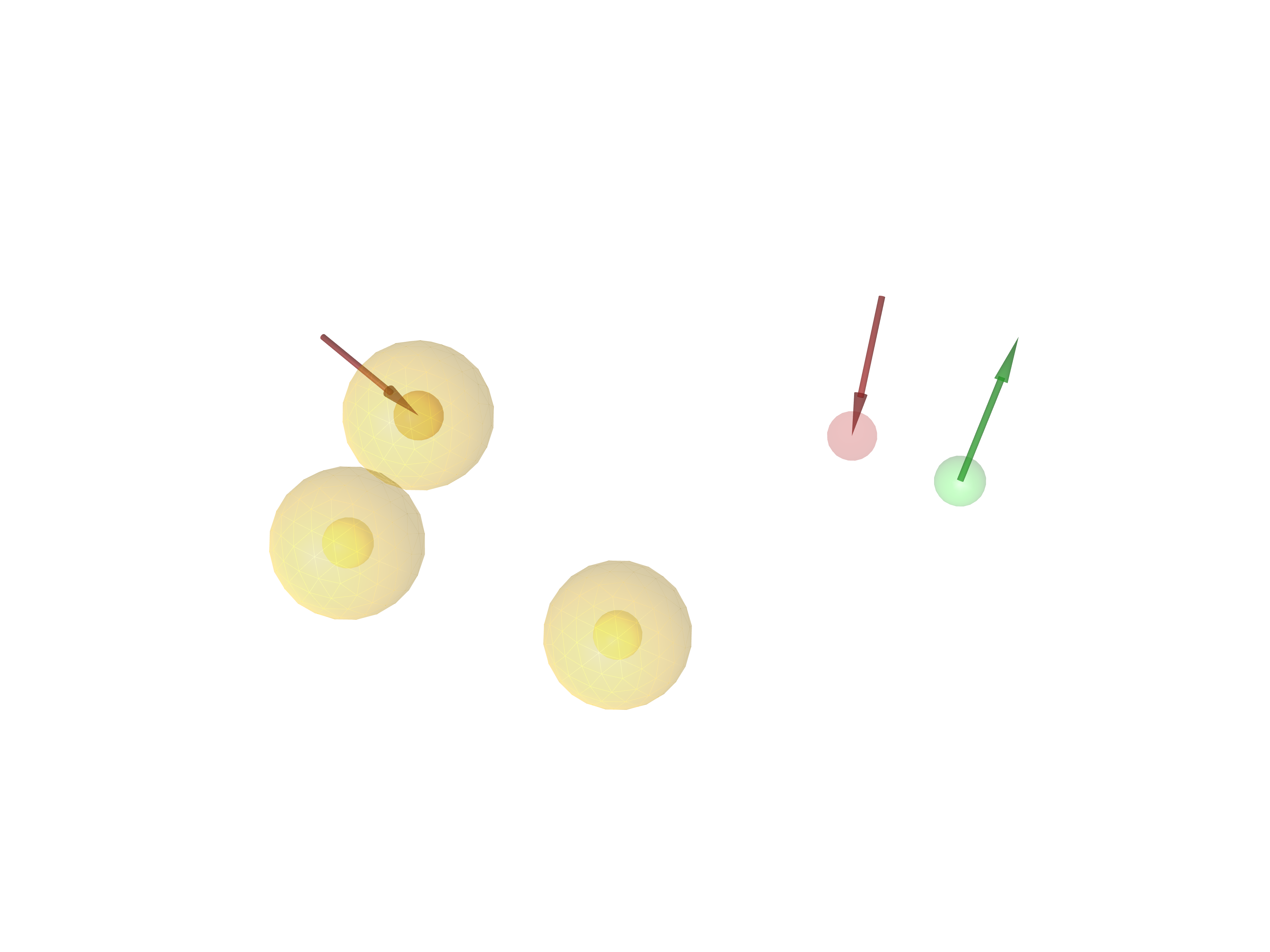

Supplement: Supplementary file 1 [file molecules-26-07201-s001.zip › pha/EGFR.png]

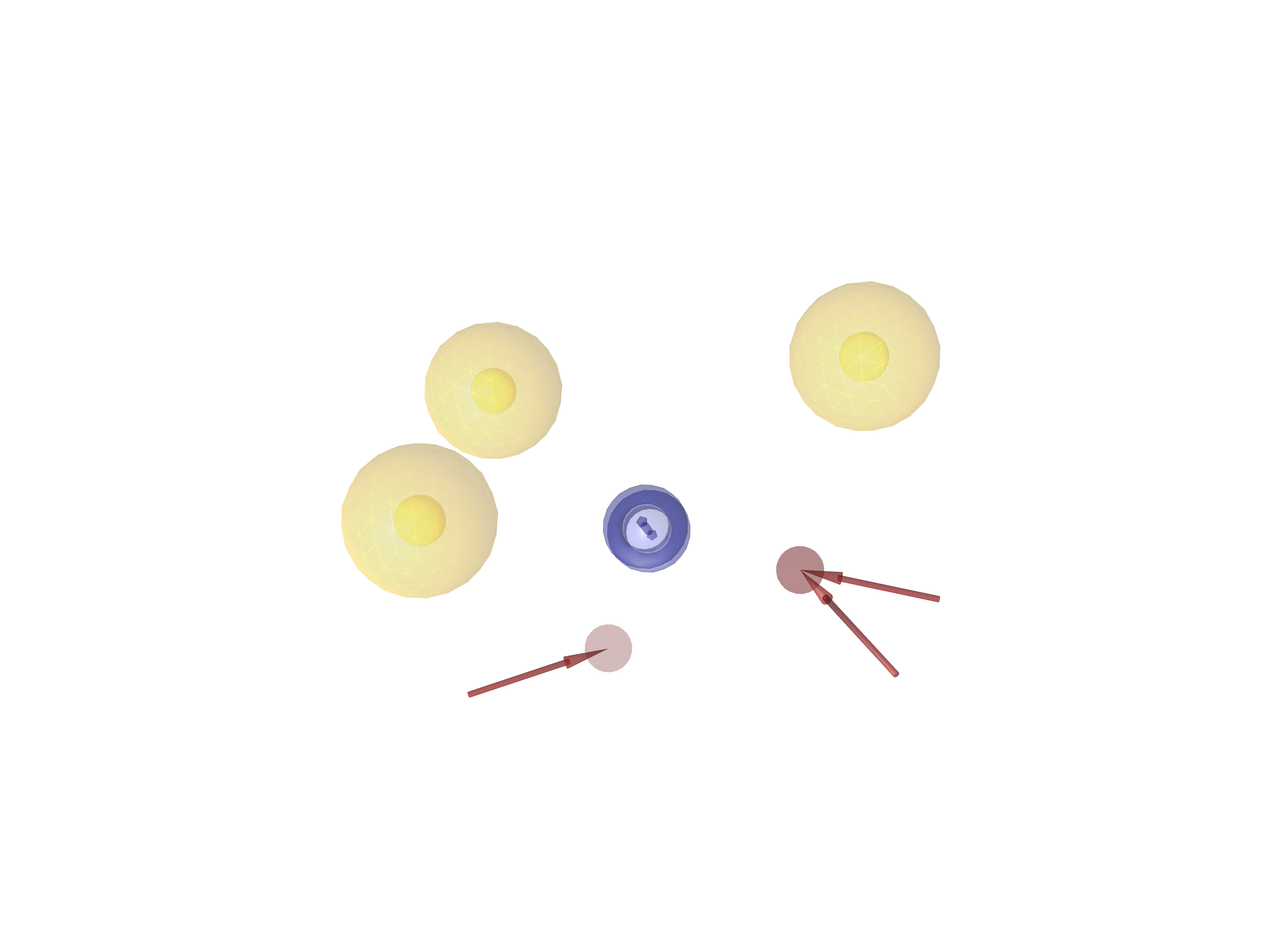

Supplement: Supplementary file 1 [file molecules-26-07201-s001.zip › pha/PDE5A.png]
